# Identification of Glutathione Peroxidase (GPX) Gene Family in *Rhodiola crenulata* and Gene Expression Analysis under Stress Conditions

**DOI:** 10.3390/ijms19113329

**Published:** 2018-10-25

**Authors:** Lipeng Zhang, Mei Wu, Deshui Yu, Yanjiao Teng, Tao Wei, Chengbin Chen, Wenqin Song

**Affiliations:** College of Life Science, Nankai University, Tianjin, 300071 China; nknanhai@163.com (L.Z.); nkwumei@126.com (M.W.); deshui_yu@mail.nankai.edu.cn (D.Y.); 2120171118@mail.nankai.edu.cn (Y.T.); weitao88888@163.com (T.W.); ccb@nankai.edu.cn (C.C.)

**Keywords:** *Rhodiola crenulata*, glutathione peroxidase, gene family, expression pattern, interaction proteins

## Abstract

Glutathione peroxidases (GPXs) are important enzymes in the glutathione-ascorbate cycle for catalyzing the reduction of H_2_O_2_ or organic hydroperoxides to water. GPXs play an essential role in plant growth and development by participating in photosynthesis, respiration, and stress tolerance. *Rhodiola crenulata* is a popular traditional Chinese medicinal plant which displays an extreme energy of tolerance to harsh alpine climate. The GPXs gene family might provide *R. crenulata* for extensively tolerance to environment stimulus. In this study, five GPX genes were isolated from *R. crenulata*. The protein amino acid sequences were analyzed by bioinformation softwares with the results that *RcGPXs* gene sequences contained three conserve cysteine residues, and the subcellular location predication were in the chloroplast, endoplasmic reticulum, or cytoplasm. Five *RcGPXs* members presented spatial and temporal specific expression with higher levels in young and green organs. And the expression patterns of *RcGPXs* in response to stresses or plant hormones were investigated by quantitative real-time PCR. In addition, the putative interaction proteins of *RcGPXs* were obtained by yeast two-hybrid with the results that *RcGPXs* could physically interact with specific proteins of multiple pathways like transcription factor, calmodulin, thioredoxin, and abscisic acid signal pathway. These results showed the regulation mechanism of *RcGPXs* were complicated and they were necessary for *R. crenulata* to adapt to the treacherous weather in highland.

## 1. Introduction

*Rhodiola crenulata* belongs to the *Rhodiola* (Crassulaceae) species which is a popular medicinal plant and has been used in traditional Chinese medicine for thousands of years [1]. The roots and rhizomes of *Rhodiola crenulata* contained several active ingredients like salidrosid, tyrosol, and their derivates which have been examined for adaptogenic and stress protection [2,3,4]. *Rhodiola crenulata* is widespread in Qinghai-Tibet Plateau (QTP) and its adjacent area with high elevations of 3500–5000 m [5]. *R. crenualta* often grows on less weathered regions with covered by gravel slopes or rock outcrops of limestones and granites [6]. The *R. crenulata* growing environment is alpine climate with characteristics of cold, drought, strong ultraviolet rays, and hypoxia [7]. The harsh circumstance made it develop slowly and promoted many changes in physiology, morphology, and genetics. In appearance, *R. crenulata* is a perennial herb which could adapt to multiple stresses [8]. In addition, *R. crenulata* employed a different carbon dioxide fixation pathway by crassulacean acid metabolism which could maintain the photosynthesis in extreme environments. However, little is known about the adaptive mechanism of genetics until now.

Plants, as sessile organisms, were susceptible to injury because they persistently accepted multiple external stimulus [9]. For the ever-changing environmental factors, abiotic stresses are the major cause to stunt plant growth by generating osmotic and oxidative stresses [10,11]. In order to survive from these stresses, plants should evolve an enormous capacity to maintain a steady state cellular circumstance by adjusting metabolism and growth [12]. Osmotic stress is induced by intracellular water deficiency, plant cells increase ctyoplasmic concentration and water retention to avoid disruption of homeostasis and ion distribution [13]. There are many molecules involved in osmotic regulation contained inorganic electrolytes like K^+^, Na^+^, Ca^2+^, and organic solutes like proline, glycine betaine, polyols, and non-reducing sugars [11]. Oxidative stress is induced by reactive oxygen species (ROS) which include oxygen ions, free radicals, and peroxides [14]. ROS are generated and accumulated at the early event of stresses and worked as a secondary messenger to trigger subsequent gene expression and defense system, however, exceeding ROS levels are damaging to cells by membrane phospholipid layers disturbation and biological molecules denaturation [15,16]. 

Considering the function and danger of ROS, a deliberate mechanism would be used to control ROS level [17]. The lifetime of ROS is mainly determined by various antioxidants like glutathione, ascorbate, and associated enzymes including superoxide dismutases (SODs), catalases (CATs), and other enzymes in glutathione-ascorbate cycle contained ascorbate peroxidases (APXs), monodehydroascorbates (MDHARs), glutathione peroxidases (GPXs), and glutathione reductases (GRs) [18]. Among them, GPXs are key enzymes for the antioxidant network. At first, unlike CATs or APX, GPXs have low substrate specificity and they are able to reduce a wide spectrum of peroxides besides H_2_O_2_ [19,20,21]. Second, some GPx isoforms exhibit the unique capacity to reduce lipid hydroperoxides thus protecting membranes oxidative stress [22,23]. At last, it has been reported that GPXs have precedence over APX to scavenge ROS under severe and persistently stressful condition [24]. With the progress of biochemical technology, the functions of GPXs have been elaborated on for crystal structures and validated through transgenic plants [19,25,26,27,28,29]. As the reports showed, GPXs were important for plants in response to abiotic stresses by multiple metabolic pathways. However, there were not any reports about *Rhodiola crenulata* GPXs. 

In recent years, RNA sequencing (RNA-seq) technology has been widely used to reveal the difference in gene transcriptional levels for many plants with the development of next generation sequencing [30]. Especially for no-model plants, these organisms were the absence of detailed genetic information or closest reference genome [31]. In order to avoid the problem of homogenetic amplification caused by non-whole genome sequencing, RNA sequencing (RNA-seq) is a quick way to obtain coding sequences, discover new genes by constructing the genomic library (cDNA) or the transcription database [32]. Based on de novo assembly technology, many Chinese medicinal plants have been carried out in transcriptome studies successfully such as *Pelargonium graveolens* [33], *Salvia miltiorrhiza* [34], *Forsythia koreana* [35], and *Panax japonicas* [36]. Conducted transcirptome analysis for different organs or developmental stages, many new genes were identified related to secondary metabolites biosynthetic pathway or cultivation and breeding process [37,38,39,40]. In addition, RNA-seq was a convenient method for intensive studies in gene families like AP2/ERF transcription factor of *Petroselinum crispum* and cytochrome P450 of *Salvia miltiorrhiza* [41,42]. 

In order to strengthen the understanding into the molecular mechanism for *R. crenulata* response to extreme environment, our laboratory conducted RNA-seq firstly, and then selected the gene family of glutathione peroxidases for further research. The cDNA sequences of *RcGPXs* were isolated from leaves of *R. crenulata*, and function analysis by bioinformation. In addition, the expression levels of *RcGPXs* for separate organs or stressful conditions were obtained by qRT-PCR. At last, physical interaction proteins of *Arabidopsis thaliana* with *RcGPXs* were investigated by yeast two-hybrid assay. 

## 2. Results

### 2.1. Identification and Alignment of GPX Genes in Rhodiola crenulata

In 2015, RNA-seq experiments were conducted for two altitudes *R. crenualta* with Rc5200 (located at 5237.2 m altitude) and Rc4800 (located at 4864.4 m altitude). According to the annotation information of assembly unigene database, five unigene protein sequences were presented high identify with plant phospholipid hydroperoxide glutathione peroxidases (Table S1). Based on the results with Blastp on NCBI, the potential open reading frames were predicted for *RcGPXs* separately. Then primers were designed according to the ORF (open reading frame) and sequences were validated by specific primer amplification products with Sanger sequencing. As the results showed, there were five unigenes annotated to GPXs, compared with eight members for *Arabidopsis thaliana* GPXs. The coding sequence (CDS) length of comp 28068 was 516 bp and encoded 171 amino acids which named *RcGPX5* (MK084908) with protein sequence shared high identity with XP_011033684.1 (*Populus euphratica* GPX4) and At3g63080.1 (*Arabidopsis thaliana* GPX5). Meanwhile, Comp 37551 was the largest gene named *RcGPX7* (MK084912) with 705 bp length encoding 234 amino acids. Comp 33811 was named *RcGPX8* (MK084909) with 513 bp length encoding 170 amino acids. Comp 35764 was named *RcGPX4* (MK084910) also with 513 bp length encoding 170 amino acids. Comp 36129 was named *RcGPX2* (MK084911) with 606 bp length encoding 201 amino acids (Table S2).

### 2.2. Bioinformation for GPXs Genes in Rhodiola Crenulata

**Figure 1:**
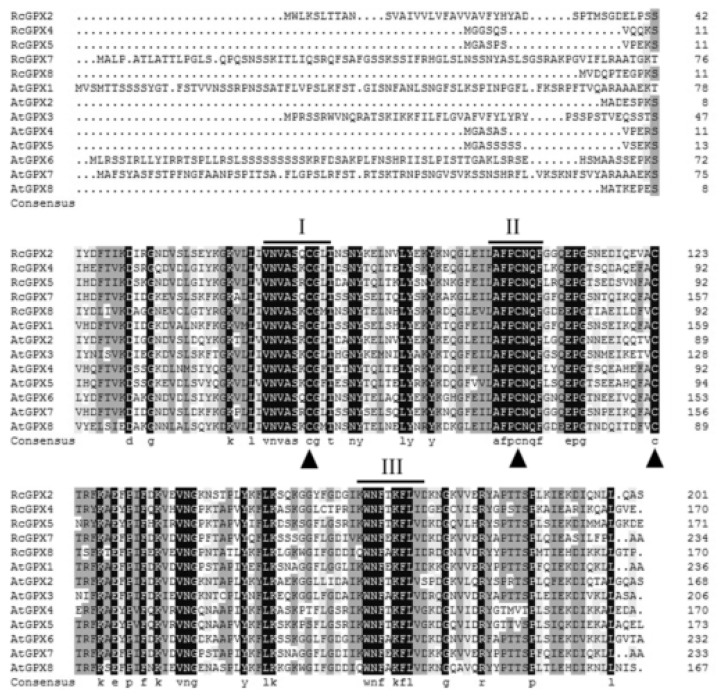
Amino acid sequences alignment of plant glutathione peroxidase by DNAMAN. The highly conserved domains were presented by line and digital alphabet I, II, and III The conserved three Cys residues of plant GPX proteins are indicated by triangles. Abbreviations of plant species: Rc, *Rhodiola crenualta*; At, *Arabidopsis thaliana*. The GPX protein sequences of *Arabidopsis thaliana* were as followed: *AtGPX1* (AT2G25080.1); *AtGPX2* (AT2G31570.1); *AtGPX3* (AT2G43350.1); *AtGPX4* (AT2G48150.1); *AtGPX5* (AT3G63080.1); *AtGPX6* (AT4G11600.1); *AtGPX7* (AT4G31870.1); *AtGPX8* (AT1G63460.1).

**Figure 2:**
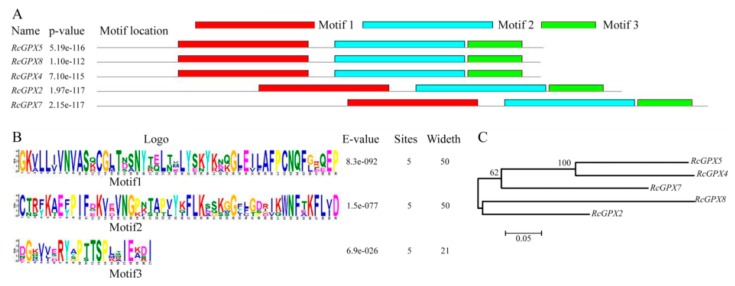
Architecture and phylogenetic tree of glutathione peroxidases of *Rhodiola crenulata*. The structures of *RcGPXs* were predicted by MEME software. Distribution of conserved domains was presented by different colors (**A**). Hidden Markov model logos obtained using MEME (**B**). The phylogenetic tree of *RcGPXs* was constructed using the neighbor-joining method of CLUSTALW by Mega 5.0, with 1000 bootstraps, and the bar indicates 0.05 substitutions per site. Each ellipse shows a clade (**C**).

**Figure 3:**
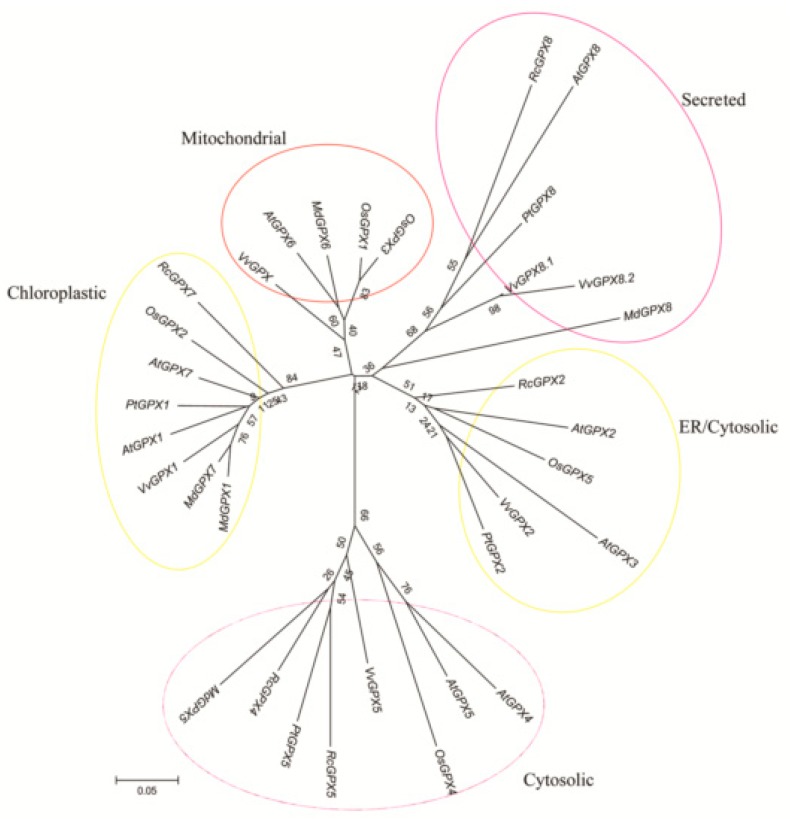
Phylogenetic analyses of thirty three plant glutathione peroxidase (GPX) proteins of *R. crenulata* and other species. The tree was constructed using the neighbor-joining method of CLUSTALW by Mega 5.0, with 1000 bootstraps, and the bar indicates 0.05 substitutions per site. Each ellipse shows a clade. The protein sequences of GPXs were followed: *AtGPX1* (AT2G25080.1); *AtGPX2* (AT2G31570.1); *AtGPX3* (AT2G43350.1); *AtGPX4* (AT2G48150.1); *AtGPX5* (AT3G63080.1); *AtGPX6* (AT4G11600.1); *AtGPX7* (AT4G31870.1); *AtGPX8* (AT1G63460.1); *VvGPX1* (XM002285528); *VvGPX2* (XM002263291); *VvGPX5* (XM002276220); *VvGPX8*.1 (XM002272900); *VvGPX8*.2 (XM010662496); *VvGPX* (XM010662503); *OsGPX1* (Os04g0556300); *OsGPX2* (Os06g0185900); *OsGPX3* (Os02g0664000); *OsGPX4* (Os03g0358100); *OsGPX5* (Os11g18170); *PtGPX1* (POPTR_0006s28120); *PtGPX2* (POPTR_0007s02160); *PtGPX5* (POPTR_0014s13490); *PtGPX8* (POPTR_0001s09280); *MdGPX1* (XP008379282.1); *MdGPX5* (XP008355452.1); *MdGPX6* (NP001280872.1); *MdGPX7* (XP008347490.1); *MdGPX8* (XP008384017.1). Plant species included: Rc (*Rhodiola crenulata*); At (*Arabidopsis thaliana*); Os (*Oryza sativa*); Pt (*Populus trichocarpa*); Vv (*Vitis vinifera*); and Md (*Malus domestica*).

Based on the protein sequences, *RcGPXs* with other GPXs of diverse resource species were shared three conserved non-SeCys acted as the catalase sites (Figure 1). By MEME software, three conserved domain were searched. As the figure presented, *RcGPXs* included three similar conserved domains (Figure 1 and Figure 2). Two Cys sites were located in VNVAS(K/R/Q/E)CGLT and LAFPCNQF domains, another was outside domain KWNF(E/T/A/S)KFLV. By online website prediction and alignment results, five *RcGPXs* were located in different subcellular positions. Comparing the model species *Arabidopsis thaliana* GPXs, *RcGPX5*, and *RcGPX4* were the same as *AtGPX4/5* co-located in cytoplasm, *RcGPX2* was likely to *AtGPX2/3* located in endoplasmic reticulum or cytoplasm, *RcGPX7* and *AtGPX1/7* were located in chloroplast, *RcGPX8* was the same with *RcGPX8* located in nucleus or cytoplasm. However, there was not a GPXs member of *R. crenulata* similar to *AtGPX6* located in mitochondria (Figure 3). The tertiary structures were predicted for *RcGPXs* by Swissmodel website (Figure S1). Using poplar GPX5 as a template which the crystal structure has been determined [19], *RcGPXs* protein tertiary structures shared similar sites and *RcGPX4*, *RcGPX5*, *RcGPX7*, and *RcGPX8* held very similar 3D structures. In addition, four of five *RcGPXs* expect *RcGPX2* were without transmembrane structures by TMHMM Server 2.0 (Table S2). 

### 2.3. Expression Levels for RcGPXs in Organs and Development Stages 

**Figure 4:**
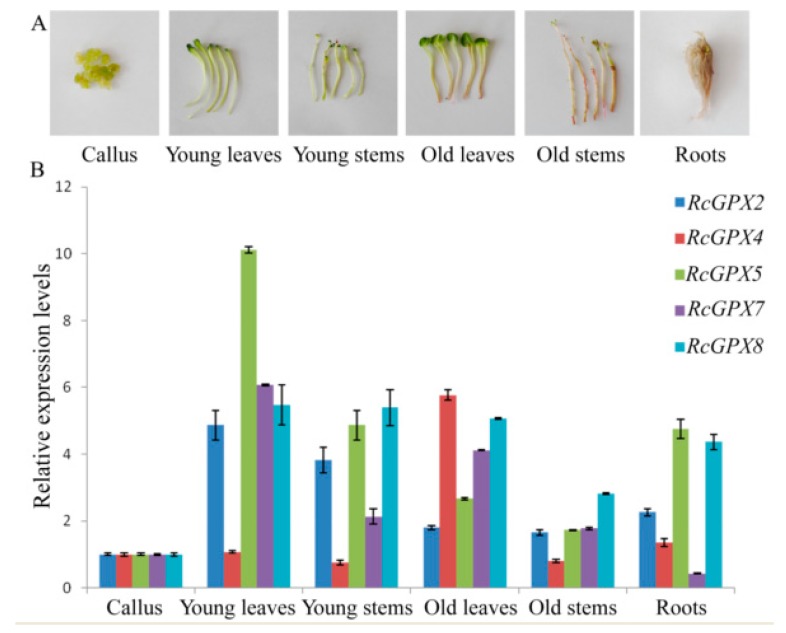
Tissue-specific expression analysis of *RcGPXs*. Six organs and tissues were used for real-time PCR (**A**). The expression levels of *RcGPXs* under different development stages or tissues were presented by colors (**B**). mRNA levels were normalized with respect to *RcGAPDH* and are expressed relative to the values of callus tissue, which were given an arbitrary value of 1. Data represent the means ± SE of at least three replicates.

In order to analyze spatial and temporal specific expression, three organs including leaves, roots, and stems and three development stages including callus, young/old leaves, and stems were selected (Figure 4). Five *RcGPXs* members were expressed in all tissues and organs above with some differences. *RcGPX4* presented parallel expression levels for tissues with the highest in old leaves. Other *RcGPXs* expression levels changed distinctly among materials with the lowest in callus and the highest in young and green tissues include leaves and stems expected *RcGPX7*. *RcGPX7* with its chloroplast location showed higher expression levels in leaves. For the overground parts, all genes showed lower expression levels in old stems whose color was red because of the longer development time. In addition, *RcGPXs* presented lower expression levels in callus. 

### 2.4. H_2_O_2_ Contents of Rhodiola Crenualta under Drought, UA, Cold and Flooding Stresses, and Expression Profiling of RcGPXs Response for These Stresses

In order to research the expression patterns of *RcGPXs* response to stresses, four treatment conditions include drought, UA (ultraviolet ray), cold and flooding were conducted for *R. crenulata* at the same developmental stage. At first, the H_2_O_2_ contents of leaves were measured to ensure the stresses were effective to plantlets (Figure S2). Compared with pre-treatment, H_2_O_2_ contents were changed distinctly with the treatment process. UA stress presented heavy damage to *R. crenulata* bodies with inducing the highest levels of H_2_O_2_ at the second day. Drought held similar tendency of UA stress in H_2_O_2_ contents with continuously increasing over the first days and then going down. However, the H_2_O_2_ contents of cold and flooding stress were reduced overall with a slightly rise over the following days and then dropping off finally. 

**Figure 5:**
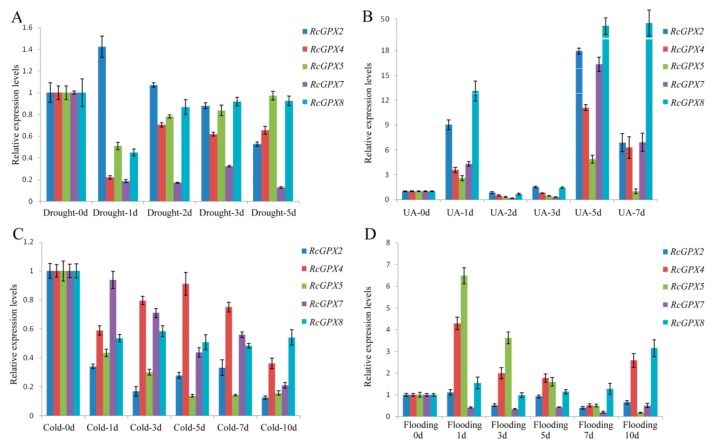
Expression patterns of *Rhodiola crenulata* glutathione peroxidase (*RcGPXs*) genes for leaves under abiotic stresses. (**A**) Drought stress was used by PEG-4000 (20%); (**B**) UA stress was used the 234 nm ultraviolet ray wavelength; (**C**) cold stress was under ice-water environment for 0 °C; (**D**) flooding stress was used to build hypoxia circumstance. The total RNA was extracted from young leaves. mRNA levels were normalized with respect to *RcGAPDH* and were expressed relative to the values at 0 day (control), which were given an arbitrary value of 1. Data represent the means ± SE of at least three replicates.

Exceeding H_2_O_2_ in plants could require high enzyme activities of GPXs, which also make an important effect on the expression levels of *RcGPXs*. However, *RcGPXs* expression levels tendencies were not completely consistent with the H_2_O_2_ contents in leaves (Figure 5). Firstly, with the highest H_2_O_2_ releasing by UA stress, all member expression levels were significantly induced by UA stress especially *RcGPX7* and *RcGPX8*. Compared with 0 day materials, *RcGPXs* expression levels were up-regulated at 1 day, while down-regulation at 2 days and 3 days, and abundantly expressed at last. For drought stress, *RcGPXs* expression levels were lower than 0 day, however up-regulated subsequently and then went to down again with the exception *RcGPX2*. *RcGPXs* response to flooding stress were very different among members. *RcGPX4*, *RcGPX5*, and *RcGPX8* could be significantly increased by flooding stress, while the expression time of *RcGPX8* was late for *RcGPX4* and *RcGPX5*. *RcGPX7* and *RcGPX2* expression levels were down-regulated at the first two days, and up-regulated at 5 days. Cold stress could decrease the expression of *RcGPXs* especially for *RcGPX2* and *RcGPX5*. *RcGPX4* and *RcGPX8* had persistently decreased expression levels and the others presented up at first and then down-regulation. 

### 2.5. The Expression Patterns of RcGPXs for Plant Hormones 

**Figure 6:**
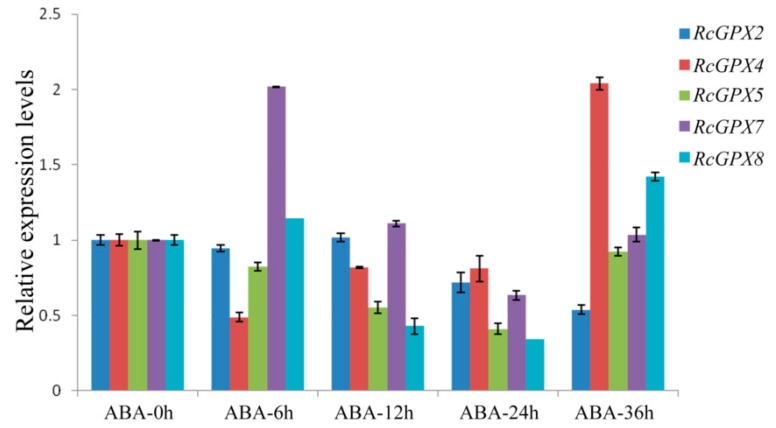
Time-course expression levels of *RcGPXs* genes for leaves under abscisic acid treatment. Plant hormone abscisic acid (ABA) was used to treat whole seedlings of *R. crenulata* at 50 μM. Five time periods of conditions were selected at 0 h, 6 h, 12 h, 24 h, and 36 h. The total RNA was extracted from young leaves. mRNA levels were normalized with respect to *RcGAPDH* and were expressed relative to the values at 0 h (control), which were given an arbitrary value of 1. Data represent the means ± SE of at least three replicates.

As is well known, plant hormones play a major role in plant development or response to stresses and GPXs expression levels were induced by multiple hormones [43,44]. With the extension of abscisic acid treatment, *RcGPX5* and *RcGPX4* were distinctly down-regulated at the early treatment stage but up-regulated at 36 h. *RcGPX2* and *RcGPX7* expression levels were increased at first and then went down. *RcGPX8* presented a complicated tendency for abscisic acid (Figure 6). In addition, the expression patterns for *RcGPXs* were changed for other hormones. Exogenous MeJA and GA_3_ treatments could negatively regulated the expression levels of *RcGPXs*, exogenous IAA treatment significantly improved the expression levels of *RcGPX4*, *RcGPX7,* and *RcGPX2* however reduced *RcGPX5* expression and made little effect on *RcGPX8* (Figure S3). These results showed that *RcGPXs* were widely regulated by plant hormones and involvement in the control of plant development. 

### 2.6. Expression Levels of RcGPXs under Heavy Metals 

**Figure 7:**
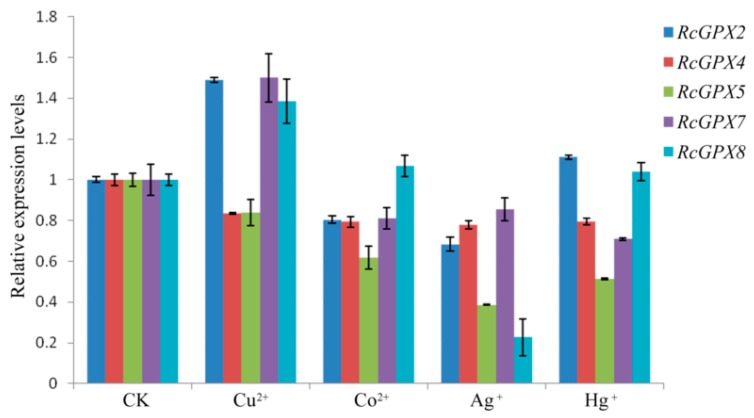
Expression levels of *RcGPXs* genes for leaves under heavy metal conditions. Heavy metal compounds were used to treat whole seedlings of *R. crenulata* for 6 h. The seedlings were exposed to MS (Murashige and Skoog) media contained different heave mental ions and the concentrations were 25 μM Hg^+^ (HgCl), 50 μM Cu^2+^ (CuSO_4_), 50 μM Co^2+^ (CoCl_2_), and 50 μM Ag^+^ (AgNO_4_). CK indicted the negative control which seedlings were exposed for MS media for 6 h. The total RNA was extracted from young leaves. mRNA levels were normalized with respect to *RcGAPDH* and were expressed relative to the CK values, which were given an arbitrary value of 1. Data represent the means ± SE of at least three replicates.

It is widely accepted that heavy metals could induce cell oxidative stress and irreversibly break down the protein or DNA molecules, furthermore, heavy metals also directly destroyed the antioxidant defense system with inactivation antioxidant enzymes or depletion of low molecular weight antioxidants [45,46]. So in order to investigate whether the expression levels of *RcGPXs* were affected by heavy metals, four ions including copper, mercury, cobalt, and silver were selected to treat the *R. crenulata* plantlets. As the results showed, exceeding copper (Cu) could induce *RcGPX2*, *RcGPX7* and *RcGPX8* expression with reduced *RcGPX4* and *RcGPX5*. Cobalt (Co) could decrease four of five *RcGPXs* expression levels while having little effect on *RcGPX8*. Exogenous silver (Ag) had a negative influence on the expression of all *RcGPXs*. In addition, for mercury (Hg) stress, *RcGPX4*, *RcGPX5,* and *RcGPX7* had decreased expression levels while *RcGPXs* presented slightly increasing levels compared to control materials (Figure 7). 

### 2.7. RcGPXs Physically Interaction with Proteins of Multiple Pathways 

**Figure 8:**
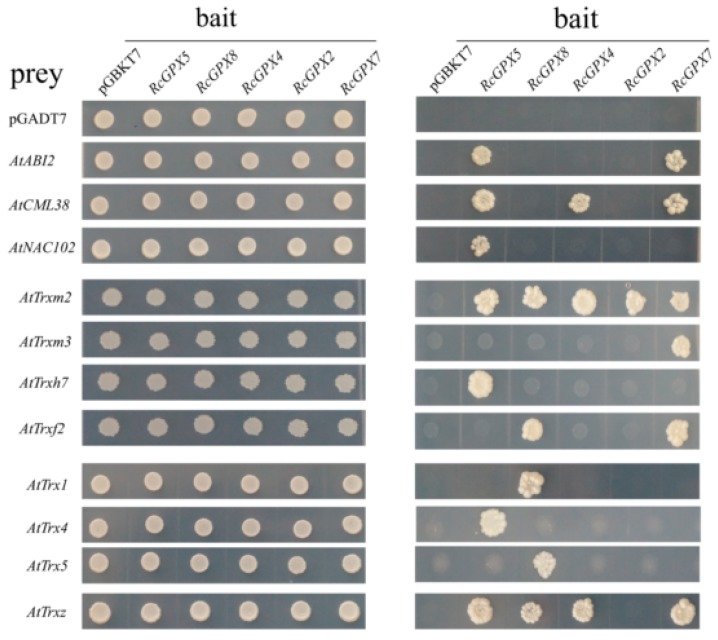
The putative interaction proteins of *RcGPXs* were investigated by yeast two—hybrid assays. Y2H gold yeast strains containing *RcGPXs* as bait and *Arabidopsis* genes as prey were grown on SD solid media lacking Trp and Leu for 5 days (**left panel**) and were assayed for reporter gene His expression by growing on SD solid media lacking Trp, Leu, and His (**right panel**). Prey and bait empty vectors were used as negative control. The *Arabidopsis* genes: *AtABI2* (AT5G57050.1); *AtCML38* (AT1G76650); *AtNAC102* (AT5G63790); *AtTrxm2* (AT4G03520.1); *AtTrxm3* (AT2G15570.2); *AtTrxh7* (AT1G59730.1); *AtTrxf2* (AT5G16400.1); *AtTrx1* (AT1G76760); *AtTrx4* (AT1G19730.1); *AtTrx5* (AT1G45145.1); and *AtTrxz* (AT3G06730.1).

It has been reported that *Arabidopsis thaliana* GPX3 participated in ABA signal pathway by physically interacted with the 2C-type protein phosphatase ABA INSENSITIVE2 (ABI2) and lesser extent with ABI1 [25]. In order to analyze the function of *RcGPXs* further, several genes of *Arabidopsis thaliana* associated with transcription factors, ethylene, calcium, and ABA signal pathways were selected to detect the interaction relationship with *RcGPXs* by yeast two-hybrid assay. As the result showed, seven ethylene syntheses key genes (*AtACS2*, *AtACS6*, *AtACS7*, *AtACS9*, *AtACO1*, *AtACO2*, *AtACO4*, and *AtACO5*) all presented no interaction with any *RcGPXs* directly. For five ABA pathway genes (*AtABI1*, *AtABI2*, *AtABI5*, *AtCYP707A2*, and *AtSNF4*), only *AtABI2* interacted with *RcGPX5* and *RcGPX7*. In addition, a calmodulin gene *AtCML38* interacted with four of five *RcGPXs* except *RcGPX8*. And a NAC (NAM/ATAF/CUC transcription factor) family member *AtNAC102* was only interacted with *RcGPX5*, however, MYB (v-myb avian myeloblastosis viral oncogene homolog) transcription factor (*AtMYB2*) and alcohol dehydrogenase (*AtADH*) showed no interaction (Figure 8). 

Thioredoxin (Trx) gene family encoding of the low weight molecules which has been confirmed to act as a substrate to provide electron donors for GPXs to catalyze H_2_O_2_ reduction and the protein model for GPXs and Trx have been calculated [19,47]. So, sixteen thioredoxin genes of *Arabidopsis thaliana* belonged to three subfamilies including y, m, and f type were chosen to investigate the protein’s relationship with *RcGPXs*. As the result showed, only half of *AtTrxs* genes presented physical interaction with one or more *RcGPXs*. For example, *AtTrxm2* interacted with all *RcGPXs*. *AtTrxz* interacted with four of five *RcGPXs* except *RcGPX2*. *AtTrx1* and *AtTrx5* presented interaction with only *RcGPX8*. *AtTrxh7* and *AtTrx4* interacted only with *RcGPX5*. *AtTrxf2* was interacted with *RcGPX8* and *RcGPX5*. In addition, *AtTrxm3* could only interact with *RcGPX7* (Figure 8).

## 3. Discussion

Despite the medical values, *R. crenulata* with other *Rhodiola* species were ideal models to research the species origin and evolution of QTP and its adjacent regions [48]. In recent years, with improvement demand for *R. crenulata*, it is becoming severely endangered due to excessive and indiscriminate exploitation with environmental destruction, furthermore, *R. crenulata* and most of this genus fail to undergo artificial cultivation [49]. In order to protect and have appropriate utilization, it needs further biological and biochemical researches for *R. crenulata*. However, because of its specific growing environment, *R. crenulata* collecting and studying were notoriously different. Consequently, little was known about *R. crenulata* with its homogenous species on genomic and genetic information [50]. Except a report about draft genome sequence, there were only several functional genes associated with salidroside synthesis pathway and DNA barcoding sequences related to genus diversity [2,49,50,51,52]. There were no reports on the mechanism of *R. crenulata* adaptation to its extreme environment. In this study, the glutathione peroxidase genes were isolated and analyzed further by bioinfomation, expression patterns, and yeast two-hybrid. This paper aimed to fill the research blank and to provide insight to its relationship with the alpine environment. 

Glutathione peroxidases (GPXs) were selected as an object for the important biological functions to cells and organisms. GPXs play a major role in ROS scavenging and biomembrane protection [24]. Consequently, ROS generated not only from oxidant damage of environment stimulus but also from the electron transfer chains in photosynthesis and respiration. It meant that GPXs extensively participated in the growth and development of plant. The expression levels of GPXs could change the H_2_O_2_ content and determine oxidant stress. Overexpression exogenous or endogenous glutathione peroxidases in plants or microorganism could provide tolerance to multipe stresses include salt, chilling, or H_2_O_2_, and methyl viologen [22,26,27,29,53]. However, knockdown or knockout expression levels of GPXs had a bad effect on development of the plant with compromised ROS scavenging system and accumulation of H_2_O_2_ [28,54]. For *RcGPXs*, our laboratory has proved overexpression *RcGPX5* transgenic *Arabidopsis thaliana* or *Salvia miltiorrhiza* presented tolerance to chilling or drought (unpublished). However members of GPXs presented divergent and redundant molecular functions partly due to the subcellular locations [44]. For seven *AtGPXs*, single mutation for any member made little influence on the shoot phenotypes, but depletion of both *AtGPX1* and *AtGPX7* which co-located in chloroplast could alter the leaf cell and chloroplast morphology [18,55]. So it might be a future decision that five *RcGPXs* members fulfilled respective functions on different biological processes, which was needed for *R. crenulata* to respond to complex environmental changes. 

Detection of transcription initiation is a necessary and widespread mechanism for plants utilized by controlling gene transcription or not and expression quantity [56,57]. Promoter sequences located in upstream of gene coding regions play a vital role in regulation genes expression. There are some specific DNA sequences named cis-regulation elements located in promoter regions which are recognized by transcription factor, plant hormones, and stresses. Using PlantCARE software [58] *Arabidopsis thaliana* and *Thellungiella salsuginea* GPXs have different numbers and types of cis-acting elements in the promoters [44]. For example, abscisic acid recognizing element ABRE (TACGTG) was investigated in *TsGPX2/4/5/7* and *AtGPX1/4/5/8*. Drought inducibility element MBS (TAACTG) existed in *TsGPX1/2/3/4/7* and *AtGPX2/5/7* [58]. In addition, promoters also contained tissue specific elements like meristem or seed specific cis-regulatory sites which determined the positions for different GPXs members function [28]. All of these elements could explain the expression patterns of GPXs response to multiple stresses treatment. In this paper, the expression patterns were different among *RcGPXs* members even for *RcGPX4* and *RcGPX5* which were the closest genes in protein sequences and had similar subcellular location. Although we don’t obtain the promoters’ information, the expression specificity for *RcGPXs* was presented by the way of tissue, development time, and stressful conditions. Therefore, it needs further studies for promoter sequences of *RcGPXs* by genome walking and transgenic technology which provided our new sights to their functions. 

As mentioned above, plant hormones or transcription factors could change the expression levels of *RcGPXs* by the cis-regulation elements in the promoter region, however, *RcGPXs* could directly interact with special proteins to participate in multiple signal pathways inversely. For example, *Arabidopsis thaliana* GPX3 physically interacted with the negative factors in ABA signal pathway to regulate plants response for drought stress [25,59]. In order to further research the function of *RcGPXs*, yeast two-hybrid assays were conducted to validate the interaction proteins from multiple pathways. Interesting, *RcGPX2* was highest identity with *AtGPX3* on protein sequence, subcellular location and transmembrane domain could not interact with *AtABI2*. However, *RcGPX5* located in cytoplasm with *RcGPX7* located in chloroplast presented interactions with *AtABI2* instead of *RcGPX4*. Furthermore, a calmodulin gene *AtCML38* and NAC transcription factor *AtNAC102* were confirmed with important hypoxia responsive sensor were also interacted with *RcGPXs* specially [60,61]. However, although it was reported that the phosphatase activity of *AtABI2* was highly sensitive to H_2_O_2_ and the redox state was coupled with *AtGPX3*, the function model for *RcGPXs* with interaction proteins needed further research. In addition, we also screened the interaction *AtTrx* protein for *RcGPXs*. As is well known, Trx proteins participate in glutathione cycle by acting as substrates for GPXs [19,62]. The plant Trx gene family contained multiply members and divided into h, f, m, o, x, and y types located in cytoplast, chloroplast, or mitochondria. However, there were no stable relations existed in Trxs and GPXs by which special GPX enzyme employed some type Trx proteins at the same subcellular location [47]. For sixteen *Arabidopsis* Trx members, eight *AtTrxs* interacted with *RcGPXs* and *AtTrxm2* could interact with all *RcGPXs*. Besides, five of eight *AtTrxs* interacted with one *RcGPX* protein. Although the results by yeast two-hybrid were not impeccable, it also enlarged the knowledge for cellular homeostasis by *RcGPXs* with Trxs. 

Different subcellular locations, expression patterns of tissue or stresses, and interaction proteins showed *RcGPXs* have extensive and complex function for *R. crenulata* growth and development. *RcGPXs* might be first in the general control of H_2_O_2_ homeostasis, and the second in linking with other pathways [25]. On the one hand, *RcGPXs* could change the contents of ROS or ratio of GSH/GSSG to create a different intracellular environment to mobilize other gene expressions, on the other hand, *RcGPXs* could participate in multiple pathways by protein-protein interaction and thiol-disulfide exchange. Consequently, *RcGPXs* plays an irreplaceable role for *R. crenulata* adapting to the treacherous weather in the highlands. 

## 4. Materials and Methods

### 4.1. Plant Materials and Stress Treatments 

In July of 2015, we cultured *R. crenulata* wild materials around Lhasa city of Tibet. Leaves of *R. crenulata* from Mila Mountain were used as explants for tissue culture and regeneration seedlings were used for subsequently experiments. For tissue-specificity analysis, four organs contained callus, roots, stems, leaves and two different developmental stages of leaves or stems were collected. Young and old leaves or stems were distinguished according to the position on the plant. With development of *Rhodiola crenulata* seedlings, the old leaves were under the young leaves with thinker in transaction and greener in color. The old stems were all in red color which were very different from young stems. The similar size and development stage of plantlets were selected to stressful treatment. For drought, the whole plantlets were immersed in MS liquid media contained 20% PEG-4000 and slightly shaken with 80 rpm. Drought stress was treated for 0 day, 1 day, 2 day, 3 day, and 5 day. For (ultraviolet ray) UA stress, the whole plantlets were exposed in UA with wave length 234 nm for 8 h and dark for 16 h one day. The plantlets were treated for 0 day, 1 day, 2 day, 3 day, 5 day and 7 day. For cold, the whole plantlets were treated with ice-water mixture for 0 °C. For hypoxia, we used blooding treatment to build the low-oxygen circumstance. The whole plantlets were placed on the bottom of glass bottle filled with sterile water, and the liquid level was 5 cm higher than the apex of seedlings. These two stresses were treated under dark for 0 day, 1 day, 3 day, 5 day, 7 day, and 10 day. After treatments, the leaves of all materials were cut carefully and divided into two parts with one was quick-freeze by liquid nitrogen with the remained fresh leaves were used to measure physiological indices. For other stresses, the whole plantlets were submerged in MS liquid media contained different heavy metals and plant hormones (25 μM Hg^+^, 50 μM Cu^2+^, 50 μM Co^2+^, 50 μM Ag^+^, 100 μM GA_3_, 100 μM IAA, 100 μM MeJA, and 50 μM ABA). The sets were slightly shaken with 80 rpm for 6 hours expect ABA. For ABA, five time periods were selected at 0 h, 6 h, 12 h, 24 h, and 36 h. Finally, the young leaves were cut and quick-freeze in liquid nitrogen. 

### 4.2. Measurement of Physiological Indices

H_2_O_2_ contents for drought, UA, cold, and hypoxia stresses were measured by 752-UV spectrophotometry. Content of H_2_O_2_ was obtained according to the method of the H_2_O_2_ Assay Kit (KeyGEN Biotech, Nanjing, China). All young leaves for these assays were come from three individuals. All data are presented as the means ± standard error (SE) of at least three replicates. The Student’s *t*-test was used to test the significance of differences compared to pre-treatment materials. And Asterisks (* and **) indicate a significant difference between the controls and transgenic plants at *p* < 0.05 and 0.01 respectively.

### 4.3. RNA Extraction and First Strand Synthesis

Total RNA for all treatments of materials was extracted using Easy RNA extract kit (Promega, Beijing, China). The quality and concentration of RNA were measured by agar gel-electrophoresis and Nano Drop 1000. Values of OD260/280 and OD 260/230 were kept in 1.8–2.2 and the concentration of RNA was higher than 200 ng/μL. Two μg RNA were used for the first strand synthesis by M-MLV (Takara, Japan) with 20 μL volumes. For any one of these treatments, three individuals were conducted separately and then cDNA were mixed into a pool. Finally, the cDNA pool was diluted to 10 times by ddH_2_O for subsequently experiments. 

### 4.4. Primers Design, PCR Amplification and Vectors Construction of Yeast Two-Hybrid 

The *RcGPXs* primers were designed by primer premier 5 according to the predicted coding sequences of assembly unigenes. To construct recombination vectors of yeast two-hybrid, the potential genes were obtained for *Arabidopsis* database (Tair: http://www.arabidopsis.org/). And primers with enzyme sites were designed according to the CDS regions. Then sequences were amplified with high fidelity thermostable DNA polymerase PrimeSTAR Max DNA Polymerase (Takara, Japan). The products were linked with pEASYT1 vectors (TransGen Biotech, Beijing, China) and sequencing by Sanger sequencing (Biotech, Shanghai, China). In addition, the recombination vectors for pGADT7 and pGBKT7 were obtained by double digestion method. Finally, the vectors were validated by PCR and sequencing. 

### 4.5. Bioinformation Analysis

The DNA sequences of *RcGPXs* were validated by Sanger sequencing again. And the protein sequences were translated according to DNA sequences. Using Blastp, *RcGPXs* protein sequences from NCBI, the homologous genes of other species were obtained and a phylogeny tree of these genes was constructed by Mega 5.0. In addition, conserve domains were predicted by MEME (http://meme-suite.org/index.html), the protein tertiary structures were predicted by Swissmodel online website (https://swissmodel.expasy.org/interactive), and the protein sequences blast was conducted by DNAMAN. And the transmembrane structures were searched by TMHMM Server 2.0 (http://www.cbs.dtu.dk/services/TMHMM/).

### 4.6. Yeast Two-Hybrid Assays

The bait and prey recombination vectors were transformed into yeast strain Y2H Gold by LiTE-PEG method. Positively yeast clones grown in SD-Trp-Leu were validated by PCR and then cultured on the solid media SD-Trp-Leu-His and the well grown clones were regarded for interaction. Then the clones were cultured in liquid media SD-Trp-Leu for 16 hours to OD_600_ 0.6–0.8 and diluted to OD_600_ 0.01 by sterile water. At last, 6 microliters was spotted on SD-Trp-Leu-His solid media, using SD-Trp-Leu solid media as control. 

### 4.7. Quantitative Real-Time PCR and Statistical Analysis

For gene expression level quantitative analysis, qRT-PCR was completed by iQ5 (Bio-Rad, Hercules, CA, USA). The reactions were 20 μL volumes contained 10 μL SYBR I (Roche, China), 10ng cDNA. Reference genes were *RcGAPDH*. Primers of *RcGPXs* for real-time PCR were designed by primer premier 5 (Table S3). The gene expression levels were calculated by the method 2^−ΔΔCt^ with three biological replicates. 

## 5. Conclusions

In conclusion, we isolated five members of the glutathione peroxidase gene family from a traditional Chinese medicinal plant *Rhodiola crenulata*. And *RcGPXs* protein sequences were analyzed by bioinformation for alignment, the conserve domain searching, sub-cellular location, and tertiary structure construction. Then the expression levels of *RcGPXs* under different tissues, abiotic stresses, and plant hormones were investigated by qRT-PCR. The results showed that *RcGPXs* were highly expressed in green and young organs, and in response to drought, cold, ultraviolet ray, and flooding stresses. Finally, several putative interaction proteins were obtained by yeast two-hybrid. *RcGPXs* members could present redundancy and divergence function in the plant body, which plays an important role for *R. crenulata* to adapt to the treacherous weather in the highlands.

## Supplementary Materials

Supplementary materials can be found at http://www.mdpi.com/1422-0067/19/11/3329/s1.
