# Supplementary figures and images for: Identification of Glutathione Peroxidase (GPX) Gene Family in Rhodiola crenulata and Gene Expression Analysis under Stress Conditions
Source: Int J Mol Sci. 2018 Oct 25;19(11):3329. doi: 10.3390/ijms19113329 (PMC6274781; doi:10.3390/ijms19113329)

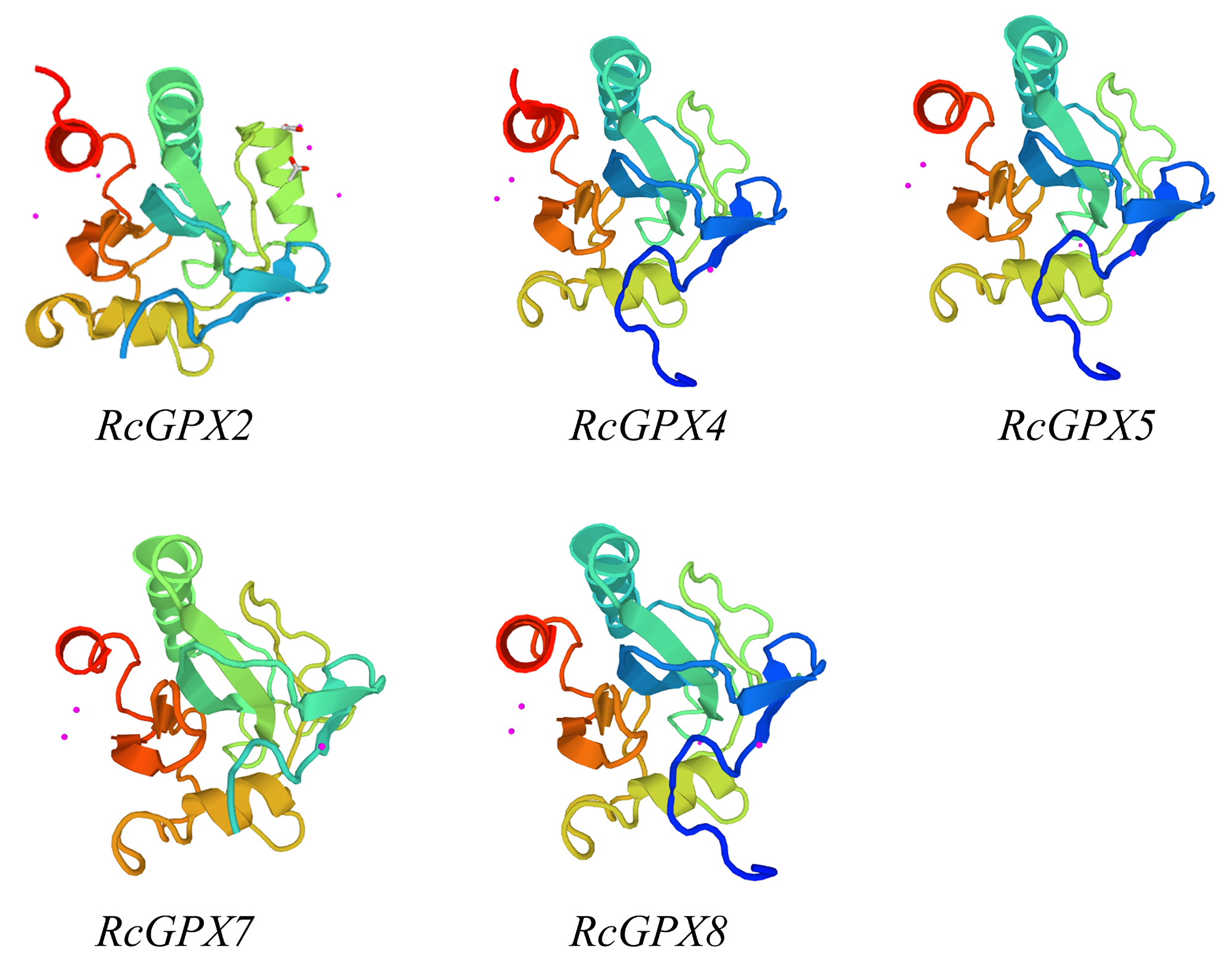

Supplement: Supplementary file 1 [file ijms-19-03329-s001.zip › supplemental/supplemental Figure 1.tif]

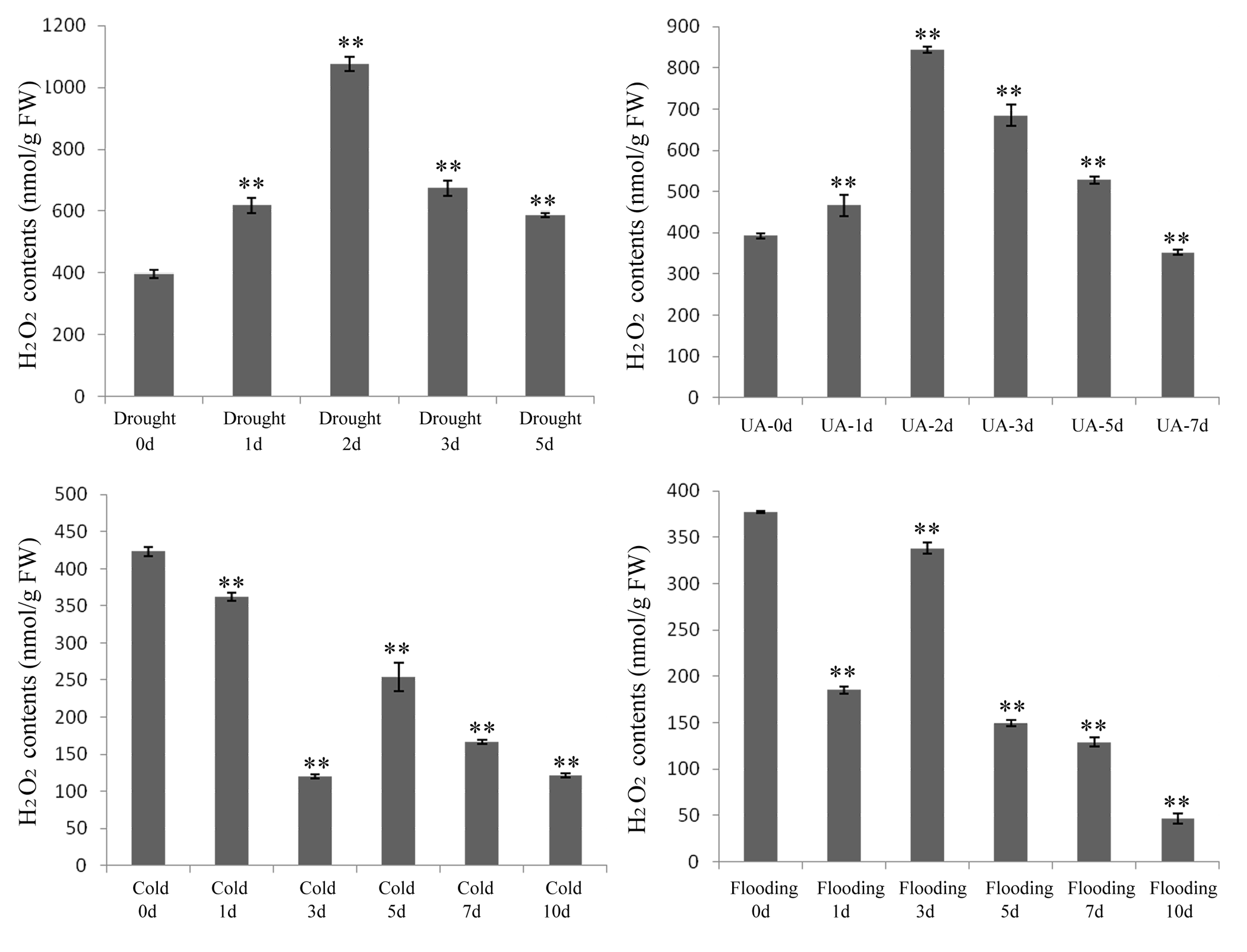

Supplement: Supplementary file 1 [file ijms-19-03329-s001.zip › supplemental/Supplemental Figure 2.tif]

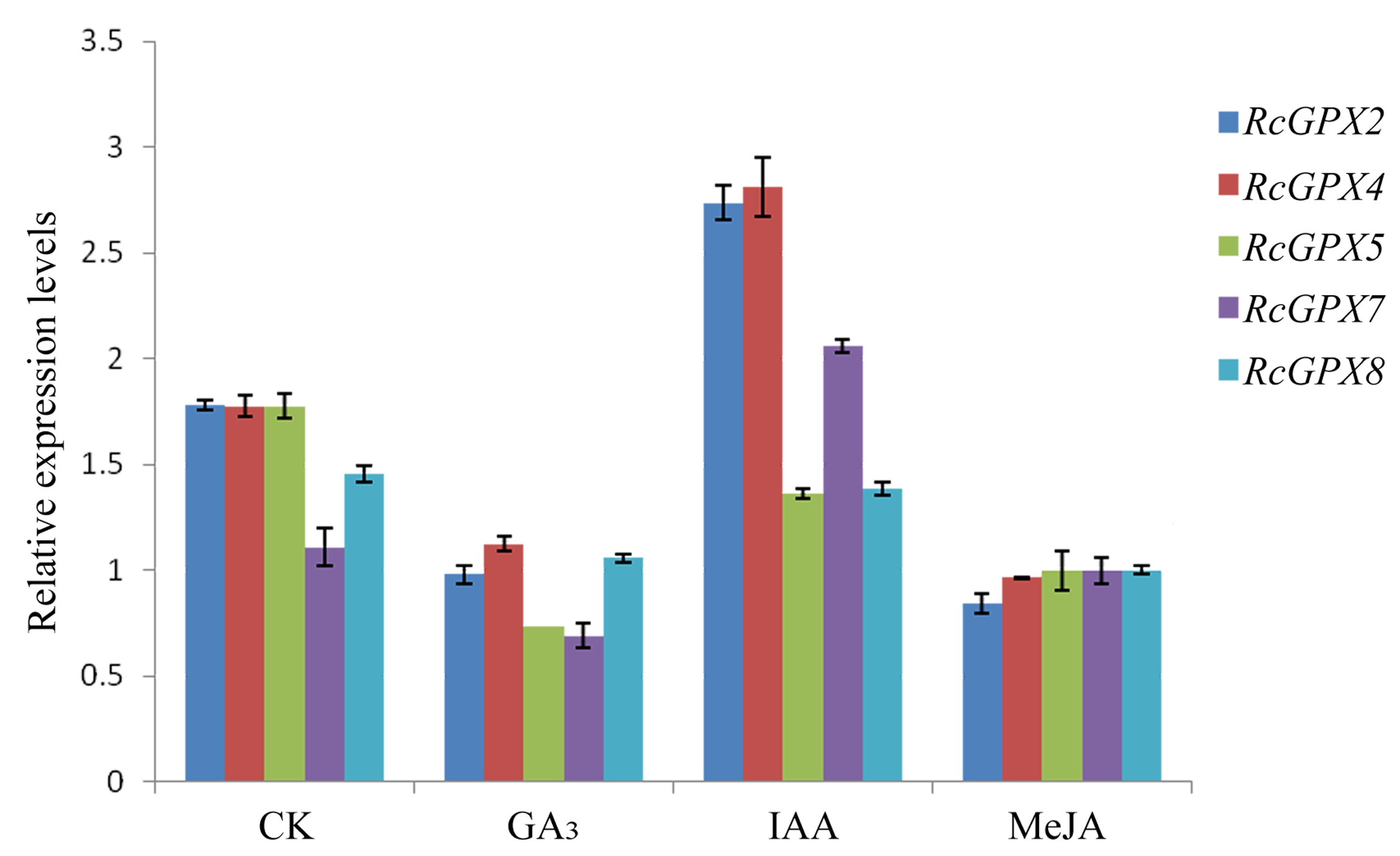

Supplement: Supplementary file 1 [file ijms-19-03329-s001.zip › supplemental/supplemental figure 3.tif]
